# MSCT findings of primary Pulmonary mucoepidermoid carcinoma

**DOI:** 10.1080/07853890.2023.2263869

**Published:** 2023-10-02

**Authors:** Ye Liu, Mengyu Liu, Yongkang Nie

**Affiliations:** Department of Radiology, General Hospital of Chinese People’s Liberation Army, P.R. China

**Keywords:** Primary Pulmonary Mucoepidermoid Carcinoma, MSCT, Lung

## Abstract

**Objectives:**

To improve diagnostic accuracy of pulmonary mucoepidermoid carcinoma (PMEC) through multi-detector computed tomography (MSCT) findings.

**Methods:**

MSCT findings of 27 histopathologically confirmed PMEC cases were retrospectively analyzed, including the location, size, margin, density, enhancement of the lesion and accompanying signs.

**Results:**

Among the 27 PMEC cases, 6 (6/27, 22.2%) were the large airway pattern, 14 were (14/27, 51.9%) the pulmonary hilum pattern, and 7 (7/27, 26.9%) were the peripheral pattern. Among those 20 cases with central pattern(6 large airway and 14 pulmonary hilum patterns), 6 presented mild enhancement, 4 moderate enhancement, 5 severe enhancement, 5 heterogeneous enhancement, and 3 with calcification. 7 cases with peripheral patterns were presented as solid pulmonary nodules and masses, 3 with severe enhancement, 1 with moderate enhancement and 3 with mild enhancement. Four cases accompanied by lymph nodal metastasis, and 7 cases with distant organ metastasis. Age(*t* = –3.132, *p* = 0.005), enlarged lymph node (*x^2^* = 9.281, *p* = 0.005), and distant metastasis(*x^2^* = 7.816, *p* = 0.008) were statistically significant in the low-grade group and high-grade group.

**Conclusions:**

MSCT images of PMEC patients demonstrated some characteristic findings, which would help improve the diagnostic accuracy of the disease.

## 1. Introduction

Pulmonary mucoepidermoid carcinoma (PMEC) is a rare, primary salivary gland-type tumor of the lung, sharing similarities with common pulmonary primary tumors of low malignant potential, but is also different from the latter. Some PMEC cases demonstrate high malignant potential and may metastasize [1]. Since PMEC has no typical clinical manifestations, and its imaging findings overlap with those of other pulmonary primary tumors, it is easily misdiagnosed. Presently, an early diagnosis and a reasonable treatment plan may be achieved through imaging and features identification to improve its prognosis. We retrospectively analyzed the clinical manifestations and MSCT image features of patients with PMEC, to achieve a better understanding and a high diagnostic accuracy of the disease.

## 2. Data and methods

### 2.1. Collection of clinical data

Twenty-seven pathologically confirmed PMEC cases were retrospectively collected in our hospital between January 2016 and December 2020. 19 were males, and 8 were females. All of the clinical history documents, including laboratory examination, and whole body image examination(such as ultrasound, CT, MRI, etc.), had been carefully reviewed in 27 cases. The primary lesions were located in the pulmonary or bronchus. The cases with findings of other sites mucoepidermoid carcinoma were excluded from this study.

### 2.2. Equipment and methods

The Philips Brilliance 256 iCT scanner was used for chest scans without and with contrast for all 27 patients. Scan parameters include tube voltage 120 kVp, automatic tube current modulation, 1.0–1.5 mm thickness thin layer reconstruction. Lung window: window width 1600HU, window level -600HU; mediastinum window: window width 400HU, window level 40HU. The scan with the contrast used a nonionic contrast medium, i.e. 70-90ml of iohexol or iopromide (300 mgI/mL), the velocity at 3.5 ml/s, and scans for the arterial phase and the venous phase was performed 25–30s and 60–65s respectively after injection of the contrast medium.

### 2.3. Imaging data analysis

Two senior chest radiologist with work experience of more than five years were recruited to observe and analyze the images on PACS workstation, including the location, size, margin, density, degree of the lesions enhancement, and existence of necrosis and calcification. In addition, they also record the condition of invading adjacent tissues, enlarged hilar and mediastinal lymph nodes, pleural effusion, distant metastasis, etc. The PMEC is subdivided into three patterns according to the lesions’ location, including the large airways pattern, where the lesions are restricted to the trachea, left and right main bronchi, and bronchi above lobar or segmental bronchi (including right intermediate bronchus); hilar pattern, where the lesion expansively grows inward and outward simultaneously, invading left and right main bronchi and the inner and outer walls of the bronchi above lobar or segmental bronchi; peripheral pattern, where the lesion is located at the peripheral bronchial branches (bronchioles below bifurcation of segmental bronchi). Levels of enhancement: mild enhancement refers to an increase of MSCT attenuation value being ≤20 HU; when 20 HU < increase of MSCT attenuation value ≤40 HU, it is moderate enhancement, and when increase of MSCT attenuation value >40 HU, it is severe enhancement.

### 2.4. Pathological examination

Pathological slicing and HE staining were performed for all 27 cases, and 2 pathology physicians with working experience of more than 5 years gave the results.

### 2.5. Statistical methods

Patients’ data were collected and sorted out using Excel 2010 software, and then handled with statistical software SPSS 24.0. A chi-squared test was used on count data, and analysis of variance was performed on measurement data following normal distribution. Post hoc pairwise comparisons of count data were performed with the Bonferroni test, and those of measurement data following normal distribution were conducted with the LSD-t test. The statistical results were deemed to have a statistically significant difference when P is less than the significance level (*α* = 0.05).

## 3. Results

### 3.1. MSCT scan findings

**Figure 1.**
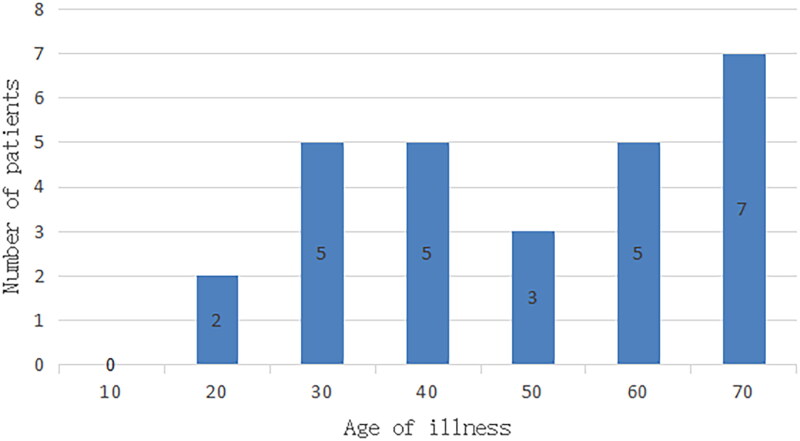
Number of patients at different onset ages.

They were aged 15–70, with the median age being 42 (Figure 1). Their clinical symptoms included coughing with sputum in 14 cases, hemoptysis in 3 cases, fever in 4 cases, chest tightness and shortness of breath in 4 cases, chest pain in 1 case, and no symptoms in 2 cases. The 27 cases in this study included 6 with large airway pattern, 14 with hilar pattern, and 7 with peripheral pattern, with lesions measuring from 13 mm to 91 mm.

**Figure 2.**
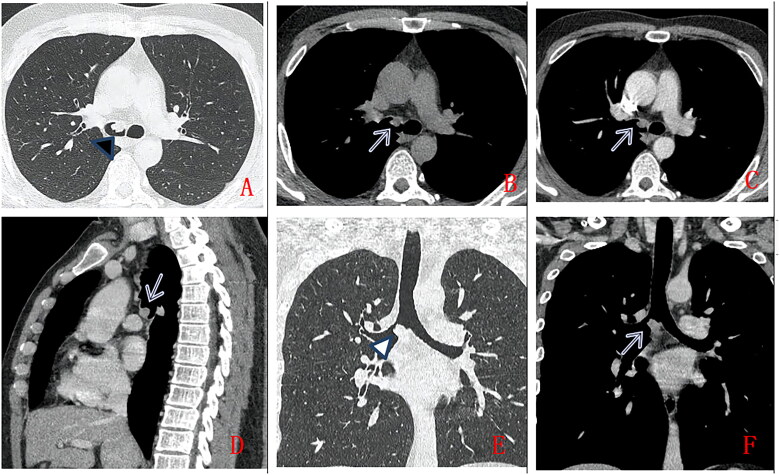
A 36-year-old man, with no clinical symptoms, chest CT scan without contrast (A.axial lung window, B.axial mediastinum window) and with contrast (C.axial, D. Sagittal, E.coronal lung window, F. coronal mediastinum window). A solid nodule in right main bronchus (as indicated by arrow), with smooth margin,uniform density, moderate enhancement. Pathologically confirmed low-grade PMEC after surgical operation.

Six cases with large airway patterns (Figure 2) include 5 in the left main bronchus and 1 in the right main bronchus The lesion is a smooth margin and well-defined, with a uniform density, CT value of approximately 25HU. Scan with contrast showed mild enhancement in 2 cases, moderate enhancement in 2 cases, and severe enhancement in 2 cases. There is 1 case with calcification, and 1 case with multiple metastases to multiple groups of lymph nodes, bone, liver and spleen.

**Figure 3.**
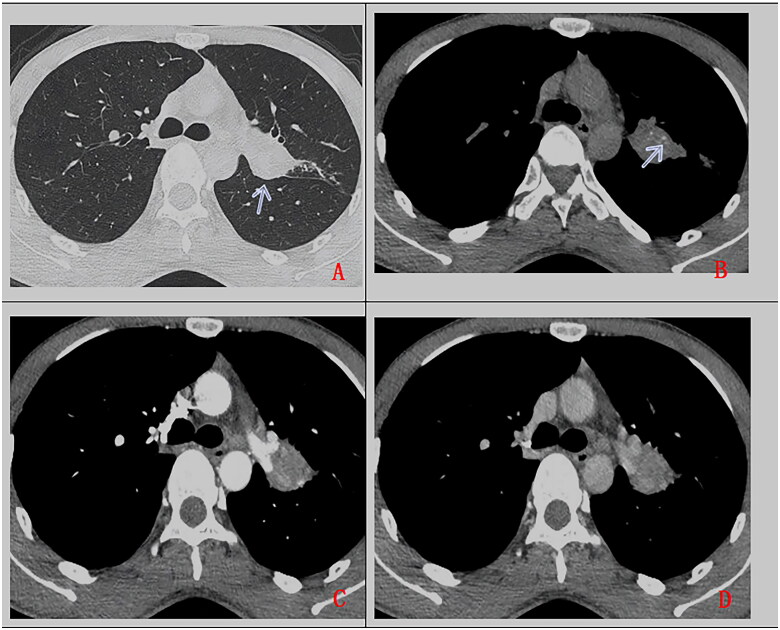
A 27-year-old man, with cough for more than 2 months. Chest CT scan without contrast (A. lung window, B. mediastinum window) and with contrast (C. artery phase, D. delayed phase). Soft tissue mass in left upper hilum (as indicated by arrow), with lobulated margin, heterogeneous density, punctuate calcification (as indicated by arrow), uneven enhancement, accompanied by adjacent bronchus obstruction. Pathologically confirmed PMEC after surgical operation.

Fourteen cases with hilar pattern (Figure 3) include 7 in the left hilum and 7 in the right hilum. In 12 cases, PMEC is accompanied by distant pulmonary atelectasis obstructive pulmonary emphysema, and bronchiectasis with mucus plugs. In the 9 cases with homogenous CT value (25HU), enhanced CT images showed mild enhancement in 3 cases, moderate enhancement in 1 case, severe enhancement in 3 cases, and uneven enhancement in 2 cases. The remaining 5 cases showed lesions of heterogeneous density(CT value 15-25HU), with uneven enhancement and severe enhancement in the solid part of the lesions. There are 2 cases with calcification, 1 case with pleural effusion, 1 case with pleural invasion, 2 cases with multiple lymph node metastasis, and 4 cases with metastases to lymph nodes, both lungs, brain, bone and liver.

**Figure 4.**
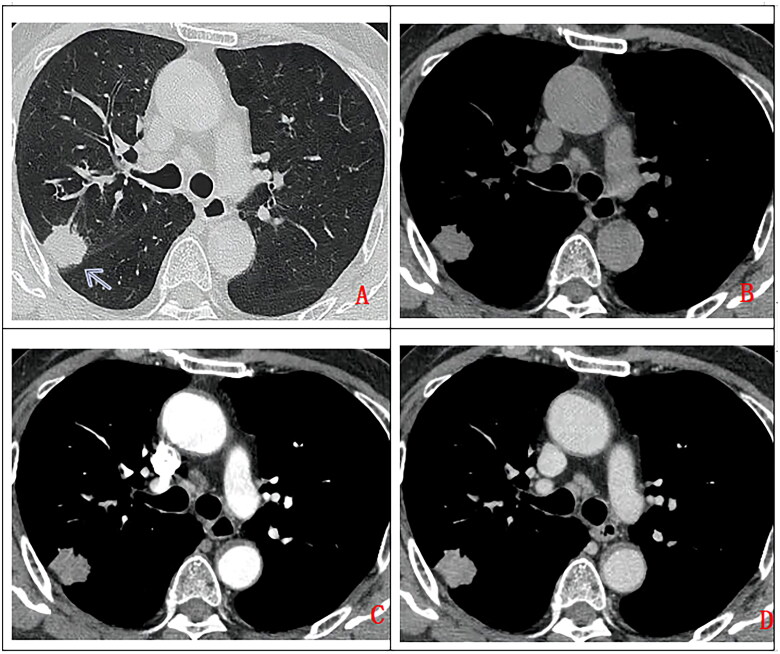
A 68-year-old man, with no clinical symptom had chest CT scan without contrast (A. lung window, B. mediastinum window) and with contrast (A. artery phase, B. Delay phase). a solitary subpleural pulmonary nodule in right upper lobe (as indicated by arrow), with lobulated margin, uniform density, moderate enhancement on contrast enhancement CT. Pathologically confirmed high-grade mucoepidermoid carcinoma after surgical operation.

Seven cases with peripheral pattern (Figure 4) include 6 cases in the right lobe and 1 case in the left lower lobe. The lesions are smooth margin, slightly lobulated. 1 case was accompanied by distant obstructive emphysema. 6 cases with homogeneous density (CT value approximately 20HU), enhanced CT images showed mild enhancement in 5 cases, and moderate enhancement in 1 case. The remaining 1 case showed heterogeneous density(CT value 15-20HU), with heterogeneous enhancement and severe enhancement in the solid part of the lesions. There was 1 case with pleural effusion, 3 cases with pleural invasion, 1 case with distant obstructive emphysema, 1 case with multiple groups of lymph nodal metastasis and 2 cases with brain metastasis.

Post hoc pairwise comparisons of the data generated the following results, with statistically significant differences existing between the three subtypes in terms of age, location of lesion (left main bronchus), pleural invasion, consolidation or pulmonary atelectasis. Firstly, the peripheral pattern has the oldest onset age (age 59.4 ± 12), while the large airway pattern has the youngest onset age (age 34.8 ± 12), and *p* = 0.031. Secondly, pleural invasion is the most common in the peripheral pattern (3/7, 42.9%), and at this point, the difference between the lung hilar pattern and the peripheral pattern is statistically significant (*p* < 0.05). Thirdly, pulmonary consolidation or atelectasis is the most common in the pulmonary hilar pattern (12/14, 86.7%) (*p* < 0.05); the difference between the hilar pattern and the peripheral pattern, and that between the hilar pattern and the large airway pattern are statistically significant (*p* < 0.05) (Table 1).

**Table 1.** Inter-group comparison of the MSCT findings of three sub-types.

Indicator		Hilar pattern	Peripheral pattern	Large airway pattern	F/χ^2^	*P*
Age		43.07 ± 17.83	59.43 ± 12.14	34.8 ± 12.72	4.041	0.031
Sex	Male	8(57.1%)	5(71.4%)	6(100.0%)	2.593	0.376
	Female	6(42.9%)	2(28.6%)	0(0.0%)		
Right upper lobe		1(7.1%)	1(14.3%)	0(0.0%)	1.130	>0.999
Right middle lobe		0(0.0%)	1(14.3%)	0(0.0%)	2.724	0.444
Right lower lobe		3(21.4%)	4(57.1%)	0(0.0%)	4.699	0.066
Left upper lobe		3(21.4%)	0(0.0%)	0(0.0%)	1.708	0.390
Left lower lobe		4(28.6%)	1(14.3%)	0(0.0%)	2.157	0.398
Right main bronchus		0(0.0%)	0(0.0%)	1(16.7%)	3.397	0.185
Right intermediate bronchus		3(21.4%)	0(0.0%)	0(0.0%)	1.708	0.390
Left main bronchus		0(0.0%)	0(0.0%)	5(83.3%)	13.823	0.000
Right main bronchus		0(0.0%)	0(0.0%)	1(16.7%)	3.397	0.185
Lobulation		0(0.0%)	2(28.6%)	1(16.7%)	4.641	0.075
Uniform density		9(64.3%)	6(85.7%)	6(100.0%)	2.157	0.398
Mild enhancement		4(28.6%)	5(71.4%)	2(33.3%)	2.030	0.452
Moderate enhancement		2(14.3%)	1(14.3%)	2(33.3%)	1.326	0.677
Severe enhancement		3(21.4%)	0(0.0%)	2(33.3%)	2.830	0.211
Uneven enhancement		5(35.7%)	1(14.3%)	0(0.0%)	2.157	0.398
Gritty calcification		2(13.3%)	0(0.0%)	1(16.7%)	1.422	0.749
Pleural effusion		1(7.1%)	1(14.3%)	2(33.3%)	3.129	0.186
Pleural invasion		0(0.0%)	3(42.9%)	0(0.0%)	6.838	0.015
Consolidation and pulmonary atelectasis		12(86.7%)	1(14.3%)	1(13.3%)	10.749	0.005
Enlarged lymph nodes		6(42.9%)	1(14.3%)	2(33.3%)	1.552	0.539
Distant metastasis		5(35.7%)	2(28.6%)	1(16.7%)	0.396	>0.999
Pathological subtypes	Low grade	6(42.9%)	1(14.3%)	4(66.7%)	2.930	0.278
	High grade	8(57.1%)	6(85.7%)	2(33.3%)		

*p* < 0.05 for inter-group comparison results.

### 2.2 Pathological results

**Figure 5.**
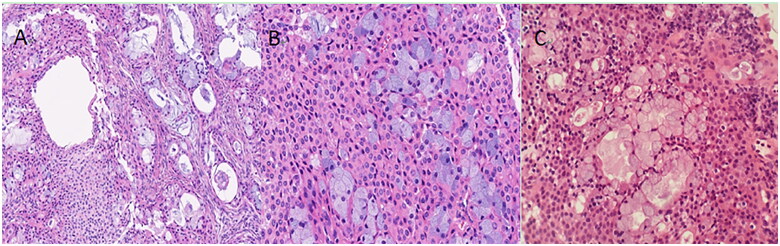
4a HE staining X100; 4b-4c HE staining X200. Pathological Slices demonstrate the tumor cells are made up of mucous cells (with lightly stained cytosol) and intermediate cells (with pink cytosol).

Of the 27 patients, 20 underwent bronchoscopy, and 7 underwent pathological examination by thoracentesis. All 27 cases were pathologically confirmed PMEC, with tumor cells comprising mucous cells and intermediate cells. Mucous cells are those with lightly stained cytosol, and intermediate cells are those with pink cytosol (Figure 5). According to WHO classification of lung cancers in 2015, MEC(mucoepidermoid carcinoma) are categorised as low-grade and high-grade based on histologic appearance [2]. In this study, 11 cases are low-grade MEC, and the remaining 16 are high-grade. Age (*t*=–3.132, *p* = 0.005), enlarged lymph node (*x*^2^=9.281, *p* = 0.005), and distant metastasis (*x*^2^=7.816, *p* = 0.008) were statistically significant in the low-grade group and high-grade group.

### 2.3. Treatment and follow-up

Of the 27 patients, 11 went through surgical treatment. 1 received pneumonectomy following interventional treatment 13 were not suitable for surgery because of distant metastasis, and chose chemotherapy. 1 was treated with radioactive particle implantation. 1 died from complications during hospitalisation. 12 patients showed no signs of metastasis or recurrence after follow-ups of 6 months to 4 years, one patient newly got adenocarcinoma, one patient experienced recurrence, and 13 patients were not in the follow-up.

## 3. Discussions

Primary salivary gland-type tumors of the lung are rare malignant tumors of the lung, including mucoepidermoid carcinoma (MEC), adenoid cystic carcinoma (ACC), epithelial-myoepithelial carcinoma (EMEC) and pleomorphic adenoma (PA) [2]. Unlike other types of primary lung tumors, primary salivary gland-type tumors of the lung are more commonly seen in the young and middle-aged population, and mainly located in large airways, but at the same time tend to be more benign [3]; MEC is the second most common type of salivary gland-type tumor after ACC.

### 3.1. Clinical manifestations

The onset of PMEC occurs between the ages of 3 and 78, most commonly affecting the population in their 30s and 40s. Of the 27 patients in this study, 20 patients were young or middle-aged, with the median age being 42. Young and middle-aged patients often show symptoms in the airways [4,5], while elder patients often show symptoms in the periphery of the lung, the difference being statistically significant and close to the report of Han[1] et al. It is usually believed that the onset of this disease shows no gender difference, but Zhuang Wu et al. [6] reported that more males were affected by the disease than females, which was consistent with the male/female composition in this study, i.e. 19 male patients and 8 female patients. The 27 cases in this study consisted of 2 asymptomatic cases and 25 symptomatic cases, with the latter accounting for 93% (25/27). Shen et al. [7] reported no apparent correlation between the incidence of the disease and smoking. The patients in this study also revealed no specific correlation between the two, with 13 patients having never smoked and 14 patients having been smoking 1 to 50 cigarettes for 10 to 50 years.

### 3.2. Imaging manifestations

PMEC is mainly of the large airway pattern and the pulmonary pattern, with lesions located at the left and right main bronchi, superior lobar or segmental bronchi. In this study, 6 cases (22%) presented nodules in the left and right main bronchi; 14 cases (52%) had masses in the left and right main bronchi and bronchi above lobar or segmental bronchi (including right intermediate bronchus); 7 cases (26%) showed a peripheral pattern. Tae et al. [8] reported that 10% of the MEC of the airway pattern occurs in the main bronchi, 75% in bronchi above lobar or segmental bronchi, and 15% in the peripheral bronchial branches. The rates are basically consistent with Tae’s report. The large airway pattern and the hilar pattern present with intraluminal nodules or masses with well-defined smooth margins, with or without accompanying obstructive emphysema or bronchus mucus plugs. The main manifestation of the peripheral pattern demonstrates smooth-margin, well-defined lobulated solitary nodules or masses. The majority of the lesions demonstrated relatively homogeneous density in our study (78%, 21/27), which is lower or equal to that of muscles of the thoracic wall, consistent with the report of Zhang et al. [9]. As to the degree of enhancement of the lesions, past reports were of different opinions[10]. Shi Jingyun[11] reported the majority of the 8 PMEC cases showed moderate or severe enhancement. Pang Junhua et al. [12] reported, most PMEC cases demonstrated mild enhancement. In our study, 10 cases (37%) presented mild enhancement, 4 cases (15%) moderate enhancement, 5 cases (19%) severe enhancement, and 8 cases (30%) obvious uneven enhancement and severe enhancement in the solid part. In MEC cases, the mucus-secreting area and the non-mucus-secreting area within the tumor have different vessel compositions and uneven distribution of such vessels, so the degree of enhancement varies[13]. Some authors [2] reported that the incidence rate of calcification in MEC can reach 50%, but this study found a lower rate of calcification in only 3 cases (11%). Fourteen cases (52%) presented obstructive pulmonary emphysema, pulmonary atelectasis, and bronchiectasis accompanied by mucus plugs distant from the lesions. Perhaps due to the growth mode of the lesion, 1 case of the peripheral type had obstructive pulmonary emphysema distant from the lesions. Maybe it was because the lesion in the bronchial branch brought about obstruction at the distant end of the affected bronchus. The peripheral pattern is more likely to have a pleural invasion, with the inter-group comparison being statistically significant. 4 cases (15%) showed metastasis to lymph nodes, 8 cases (30%) showed distant metastasis to multiple organs, and 2 cases presented pleural effusion (See Table 2).

**Table 2.** Comparison of clinical data and CT findings between low-grade and high-grade groups of pulmonary mucoepidermoid carcinoma patients.

Group	Age (*x̅*±*s*, years)	Male: Femal	pattern [*n*(%)]			Uniform Density [n(%)]	Enhancement degree [*n*(%)]				Punctuate calcification [*n*(%)]	Extrapulmonary Manifestations[*n*(%)]	
			Large airway pattern	Hilar pulmonis pattern	Peripheral pattern		Mild	Moderate	Marked	Uneven		Enlarged lymph node	Distant metastasis
Low-grade group	34.64 ± 15.81	6: 5	4 (36.4)	6 (54.5)	1 (9.1)	10 (90.9)	4 (36.4)	4 (36.4)	4 (36.4)	3 (27.3)	2 (18.2)	0(0)	0(0)
High-grade group	53.44 ± 14.60	13: 3	2 (12.5)	8 (50)	6 (37.5)	11 (68.8)	9 (56.3)	2 (12.5)	2 (12.5)	3 (18.8)	1(6.3)	9(56.3)	8(50)
*χ^2^*/*t*	-3.132	2.229	2.148	0.054	2.739	1.852	1.033	2.148	2.148	0.274	0.940	9.281	7.816
*P*	0.005	0.206	0.187	>0.999	0.183	0.350	0.440	0.187	0.187	0.662	0.549	0.003	0.008

Bronchoscopy is the most important diagnostic technique and is usually used to confirm cytological diagnosis before surgery. In this study, 20 cases were confirmed through bronchoscopy, and only 7 through examination by thoracentesis, which is mostly used in PMEC of the periphery.

### 3.3. Pathological features

The pathological features divide PMEC into two types, low-grade and high-grade. Low-grade carcinomas are more common and are mainly made up of cystic substances. There is calcification possibly related to the deposition of calcium salts caused by incomplete absorption of mucus secreted by mucous cells. Han et al. [14] also reported that the calcification of mucous secretion in PMEC was usually seen in patients with intermediately and highly differentiated tumor cells, that is, in low-grade MEC patients. In this study, only 3 cases presented calcification, and they were all low-grade carcinomas, which means such calcification could possibly be a predictor of the lesser malignancy of PMEC tumors [15]. High-grade carcinomas are rare, and they are made up of intermediate cells and epidermoid cells, with few mucous cells. Because of the particularity of the case source (most of the patients who come to our hospital for examination and treatment are difficult cases or rare cases), the data collected are biased, and the results are inconsistent with the literature reports. The tumors are mostly solid, featuring obvious cellular atypia, active karyokinesis, easily visible necrotic lesions, and common metastases to the lung hilum and regional lymph nodes [16]. Though PMEC progresses slowly, it is, in terms of biological features, different from adenoid cystic carcinoma and carcinoid tumor which tend to be a lesser malignancy, so it will finally develop local invasion and metastasis to lymph nodes, showing progressive invasion [8]. Among the 16 high-grade PMEC cases in this study, 13 cases experienced metastases to the lung hilum, mediastinal lymph nodes and other visceral organs, representing a higher rate than in other cases. In consideration of the limited sample number in this study, this conclusion may be biased. Some researchers reported that bronchiectasis accompanied by distant mucus plugs, which had been confirmed as the result of the large quantity of mucus secreted by the airway mucous cells [17]. Fourteen lung hilum-type PMEC cases in this study manifested bronchiectasis accompanied by the formation of mucus plugs distant from the lesions, basically consistent with the aforementioned report.

### 3.4. Treatment and prognosis

PMEC is mainly treated by pneumectomy. Among the 12 patients who underwent surgical treatment, 6 patients reported no abnormality in the follow-up, 1 patient newly got adenocarcinoma after a one-year follow-up, 1 patient had a recurrence after a four-year follow-up, and 4 patients were not in the follow-up. The radiotherapy and chemotherapy were relatively effective, with 14 patients treated by radiotherapy and chemotherapy reporting no abnormality in the follow-up. The prognosis was related to the patient’s age, location of the lesion and malignancy of the disease. After thorough removal of the carcinoma, low-grade MEC patients can be cured, but high-grade MEC patients are likely to suffer from distant metastasis, leading to poor prognosis.

This study carried several limitations. First, the number of PMEC cases we have collected is relatively small, which is likely to lead to statistical bias. Second, it is difficult to avoid the existence of selection bias due to the retrospective study. Third, 13 patients were not followed up.

## 4. Conclusion

In summary, PMEC demonstrates certain features on MSCT images, such as solitary and smooth nodules or masses in the large airways and pulmonary hilum, with uniform density on plain CT scan, and moderate or severe enhancement in the solid part on contrast-enhanced CT images in young people. It will be helpful to improve the diagnostic accuracy of the disease.

## Data Availability

The raw data supporting the conclusions of this article will be made available by the authors. All relevant data in this study are freely available to any scientist wishing to use them for non-commercial purposes, without breaching participant confidentiality.
